# Predicting Polysomnography Parameters from Anthropometric Features and Breathing Sounds Recorded during Wakefulness

**DOI:** 10.3390/diagnostics11050905

**Published:** 2021-05-19

**Authors:** Ahmed Elwali, Zahra Moussavi

**Affiliations:** 1 Biomedical Engineering Graduate Program, University of Manitoba, Winnipeg, MB R3T 5V6, Canada; Zahra.Moussavi@umanitoba.ca; 2 Electrical and Computer Engineering Department, University of Manitoba, Winnipeg, MB R3T 5V6, Canada

**Keywords:** obstructive sleep apnea, screening, machine learning, correlation, trachea, sleep report

## Abstract

Background: The apnea/hypopnea index (AHI) is the primary outcome of a polysomnography assessment (PSG) for determining obstructive sleep apnea (OSA) severity. However, other OSA severity parameters (i.e., total arousal index, mean oxygen saturation (SpO2%), etc.) are crucial for a full diagnosis of OSA and deciding on a treatment option. PSG assessments and home sleep tests measure these parameters, but there is no screening tool to estimate or predict the OSA severity parameters other than the AHI. In this study, we investigated whether a combination of breathing sounds recorded during wakefulness and anthropometric features could be predictive of PSG parameters. Methods: Anthropometric information and five tracheal breathing sound cycles were recorded during wakefulness from 145 individuals referred to an overnight PSG study. The dataset was divided into training, validation, and blind testing datasets. Spectral and bispectral features of the sounds were evaluated to run correlation and classification analyses with the PSG parameters collected from the PSG sleep reports. Results: Many sound and anthropometric features had significant correlations (up to 0.56) with PSG parameters. Using combinations of sound and anthropometric features in a bilinear model for each PSG parameter resulted in correlation coefficients up to 0.84. Using the evaluated models for classification with a two-class random-forest classifier resulted in a blind testing classification accuracy up to 88.8% for predicting the key PSG parameters such as arousal index. Conclusions: These results add new value to the current OSA screening tools and provide a new promising possibility for predicting PSG parameters using only a few seconds of breathing sounds recorded during wakefulness without conducting an overnight PSG study.

## 1. Introduction

Obstructive sleep apnea (OSA) is a common disorder characterized by repetitive partial or complete episodes of airflow obstruction that can result in intermittent hypoxia, transient hypercapnia, and arousals from sleep. Commonly, the severity of OSA is mainly determined by the apnea/hypopnea index (AHI), which represents the number of apnea/hypopnea events per hour of sleep. Based on the AHI values for adults, the severity of OSA is categorized into no-OSA (AHI < 5), mild (5 ≤ AHI < 15), moderate (15 ≤ AHI < 30), and severe OSA (AHI ≥ 30) [1]. However, many other parameters are measured during overnight polysomnography (PSG), the gold standard of sleep apnea detection.

Approximately 1 billion of the world’s population between the ages of 30 and 69 years are estimated to have OSA [2]; yet, it is still underdiagnosed [3]. OSA diagnosis and recommending a treatment option using the gold-standard PSG are expensive, laborious, and time-consuming; also, PSG is not available in remote areas. There are portable PSG monitoring devices that can be lent to individuals to assess their OSA overnight at home. However, to recommend a treatment option, there is still a need for a full PSG study at a sleep center. There are questionnaires such as STOP-BANG or the Epworth sleepiness score for a quick OSA screening, but they lack objectivity and have very poor specificity (~20) [4,5], which can increase the waiting list and lead to unnecessary referrals to sleep centers for full PSG studies; hence, adding unnecessary costs to the health care system. On the other hand, undiagnosed OSA, in particular, increases the perioperative morbidity and mortality risks for OSA patients undergoing surgery requiring full anesthesia [6]. Therefore, there is a need for a quick, objective screening tool to aid fast and accurate results with high sensitivity and specificity of OSA detection.

Our previous studies [7,8] have shown the ability to predict the severity of OSA, based on AHI, by analyzing a few minutes of breathing sounds recorded during wakefulness. We also demonstrated the effects of anthropometric information on the classification process [7,9]. Our latest OSA screening algorithm, called AWakeOSA [8], resulted in a blind testing accuracy >81% with a balanced specificity and sensitivity with a threshold of AHI ≥ 15. The threshold of AHI ≥ 15 was used because it is the most clinically accepted threshold to identify individuals with OSA who might benefit from the treatment [10].

Currently, AHI is the primary PSG parameter for determining an individual’s OSA severity. However, other PSG parameters, such as total time of sleep (TST), total arousal index, and mean oxygen saturation (SpO2%) of TST, are also important for a full clinical diagnosis of OSA [11]. Using the AHI parameter alone neglects events’ duration, oxygen desaturation depth, the difference of impact between apneic and hypopnic events, etc. Nevertheless, knowing these parameters helps with understanding one’s OSA pathology and choosing the proper treatment [12].

The upper airway (UA) of OSA patients is characterized by a structural deformation, narrower cross-sectional area, and more regional stiffness compared to those of healthy individuals [13,14]. These changes of the UA affect breathing sounds as they are generated by the flow of air in the UA [15]. To the best of our knowledge, there is no published work predicting PSG parameters, other than AHI, using a screening tool conducted during wakefulness. Therefore, this study for the first time demonstrates the proof of concept of a technique to predict PSG parameters representing OSA severity without a sleep study and only using a few tracheal breathing sounds recorded during wakefulness and the person’s anthropometric information such as neck circumference (NC), body mass index (BMI), etc. Hence, we hypothesize that breathing sounds characteristics (features) would be representative of the OSA severity. More specifically, we hypothesize that tracheal breathing sounds have features capable of predicting PSG parameters such as total arousal index, mean SpO2%, etc. that are related to OSA severity.

## 2. Materials and Methods

**Figure 1:**
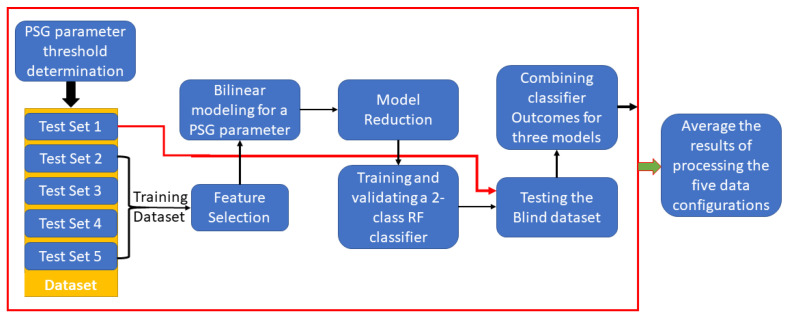
The general flow chart of the process.

We attempted to solve a two-class classification problem to predict each PSG parameter separately. The data of each PSG parameter was divided into two severity classes/groups based on a threshold (see Section 2.3). One of the two groups represented the normal case, while the other represented the abnormal case. For each PSG parameter, a classification threshold was evaluated, distinctive features were selected, and a model was created using anthropomorphic parameters and breathing sound features. Then the model was used in a classification approach to predict the PSG parameter’s severity group (normal or abnormal); violating the threshold (in the abnormal group) implied a high risk of OSA severity; check Figure 1 for the general flow of the process.

### 2.1. Study Population

The data for this study were adopted from our previous study [8]. During wakefulness, about 1–2 h prior to conducting the PSG study at Sleep Disorders Center in Misericordia Health Centre (Winnipeg, Canada), breathing sound recording was performed. Patients diagnosed with any other respiratory diseases were excluded. We obtained the PSG study report of the participants after a sleep technician completed their overnight PSG assessment analysis. All study participants signed an informed consent approved by the Biomedical Research Ethics Board of the University of Manitoba (approved on 27 October 2011) prior to the breathing experiment. Out of the 199 individuals in our previous study, data of the 145 (60 females) participants who had a complete PSG report were considered in this study.

### 2.2. Procedure and Measurements

Prior to recording, we collected the height, weight, age, Mallampati score (MpS), neck circumference, and smoking history of each participant. For simplification, the AHI of 15 was used to represent the anthropometric parameters in the two severity groups. We checked whether the data followed the normal distribution for each anthropometric parameter by using the Lilliefors test. The two severity groups were only matched in terms of age, and a *t*-test was used.

All participants conducted a full overnight PSG study. The Sleep Centre used the Natus Sandman PSG collection system (Natus Canada, Oakville, ON, Canada), which recorded 6 channels of electroencephalogram signals, chin and leg electromyography signals, electrooculography signals, electrocardiogram signals, snoring sounds, thorax and abdomen efforts signals, air flow and pressure signals, pulse and SpO2 signals, transcutaneous oxygen pressure signals, and video image for body position detection. A full overnight PSG study reports 79 different parameters reflecting on one’s OSA severity; they are listed in Table A1 of the Appendix A. The data in these reports were used for verification of our predictive modeling outcomes.

#### Tracheal Breathing Sounds

The tracheal breathing sounds were recorded using a Sony microphone (ECM77B—Advance Electronics, Winnipeg, MB, Canada) during wakefulness in a supine position. The microphone was placed over the suprasternal notch of the trachea using a double-sided adhesive ring tape. The acoustic signals were amplified, band-pass filtered (0.05–5000 Hz, Biopac DA100C—BIOPAC Systems Inc., Goleta, CA, USA), and sampled at 10,240 Hz. Participants were instructed to breathe five deep breaths through their nose, then another five cycles through their mouth; for more details on the recording protocol and the preprocessing stage, see [15]. The spectra and bi-spectra of the signals were estimated using the welsh method [16] and an indirect class conventional bispectrum estimator [17], respectively. The breathing sounds’ characteristic features, used to predict AHI in our previous study [8], were also used to predict the PSG parameters (other than AHI) in this study (see Table 1 for the list of features).

### 2.3. Threshold Determination, Data Preparation, and Feature Selection

Classification threshold determination for each PSG Parameter—80% of the data were used in the threshold determination process. In order to find a proper threshold for each PSG parameter, we scaled the PSG parameter’s values to be normally distributed with an absolute skewness less than 0.5. Basically, if the skewness was ≤−0.5, the PSG parameter was squared, and if the skewness was ≥0.5, the logarithm scale of the PSG parameter was used. Then, the overall range of a PSG parameter’s values was divided into 20 equal divisions to form threshold candidates for the classification process. For example, the TST range was between 0.5 and 7 h, and it had a skewness <0.5; thus, it did not require scaling. With the above 20 equal divisions, the potential classification thresholds were found as 0.825, 1.15, 1.475, … 6.675.

**Figure 2:**
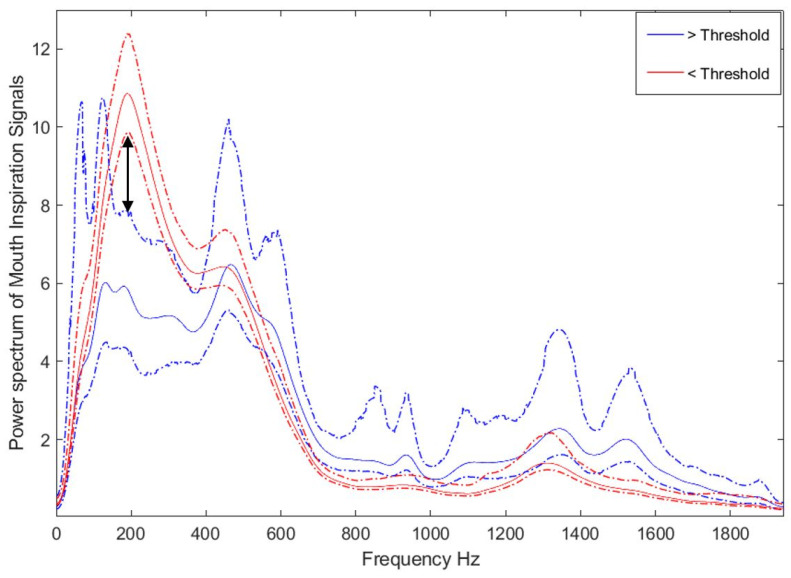
The average power spectrum of the signal recorded from mouth inspiration. Dotted lines represent the 95% confidence interval. Red and blue color curves represent the groups with more and less than a threshold, respectively. The arrow shows a large gap beyond the confidence interval between the two groups.

Classification threshold selection—For each threshold candidate of a PSG parameter, the power spectra of the sound signals of those with PSG parameter values less than the candidate threshold were grouped together, and the same was done for those with values higher than the candidate threshold. The average curve for each of these two groups was calculated with its 95% confidence interval. At some frequency bandwidths, there was no overlap between the curves of the two groups; see Figure 2 for the gaps (i.e., 150–300 Hz, 720–900 Hz, 1050–1120 Hz, and 1450–1700 Hz). Then, the average absolute difference between the 95% confidence intervals’ boundaries at the non-overlapped regions was evaluated. This process was repeated for each candidate threshold. Then, the threshold with the largest gap between the two groups and a balanced number of individuals (at least 20 individuals per group) was selected as an optimum candidate threshold. If there was more than one selected threshold for a PSG parameter, the one with a physiological meaning was selected. These three criteria to choose a threshold reduced the number of PSG parameters to be predicted from 78 to 51.

**Figure 3:**
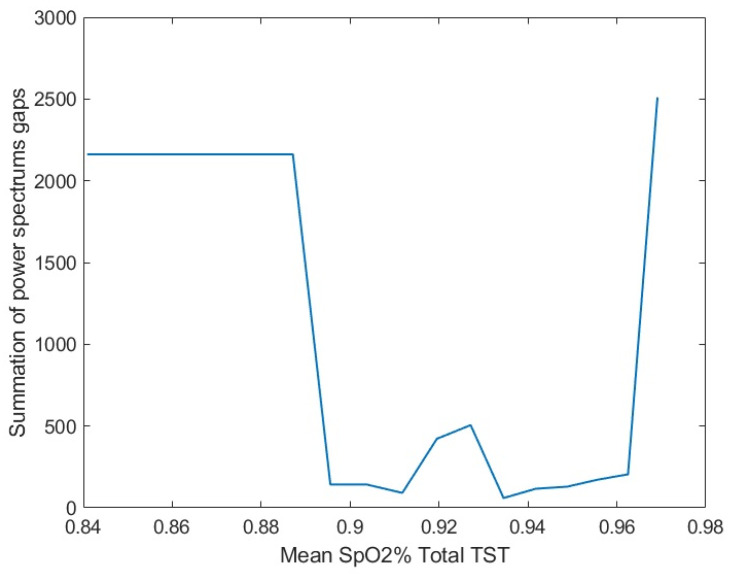
The average gap values among different breathing maneuvers for different thresholds of the mean SpO2% total TST.

Figure 3 shows an example of the gap values for different thresholds for a PSG parameter (i.e., the mean SpO2% during total TST); thresholds <92% or >95% had high average gap values, but the number of individuals per group was less than 20. The physiological meanings for different PSG parameters were discussed in the literature, in particular, the percentage of stages III and REM of sleep [18], total arousal index [19], mean SpO2% [20], and oxygen desaturation (de-SpO2) index [21].

Dataset segmentation—The dataset was divided into five folds of blind testing datasets; one-fold consisted of 20% of the data above the PSG parameter threshold and 20% of the data below the threshold. Taking the 20% above and below the threshold guaranteed no significant difference between the testing and training datasets, and it also made the two datasets comparable with the same severity level. Each fold of testing (20% of the data) with its corresponding training and validation dataset (80% of the whole dataset) were separately analyzed. Herein, we call them the five data configurations.

Feature selection—The sound features were adopted from different anthropometric groups of features used in our previous work [8]; therefore, some features might be highly correlated with one another and need to be filtered. Thus, for a PSG parameter, the correlation coefficient (CC) between every pair of sound features was calculated; if the CC was ≥0.95, we calculated the CC between each of the two features and the PSG parameter, and the feature with the lowest CC was subsequently removed. This approach guarantees removing similar features. Then, each feature’s data were divided into two groups based on the selected PSG threshold. The outliers for each feature were identified in each PSG severity group and then removed [22]; this process is delineated in our previous work [8]. The significance between the two severity groups was calculated, and only features with a *p*-value ≤ 0.05 were selected. Therefore, each PSG parameter has its own significant set of features. These processes were separately applied to the five data configurations.

### 2.4. Bilinear Modeling and Correlation Analysis

A bilinear polynomial model of three variables (a three-feature combination of sound and anthropometric features) was generated for each PSG parameter; see Equation (1) for the polynomial model (X, Y, and Z are three features, and a is a constant). We did not use higher-order models to avoid overfitting due to the small sample size. For simplicity, we mentioned only the methodology using the three-feature combination, but the presented results are for three-, four-, and five-feature combinations. The model was created from each three-feature combination using the multiple linear regression function in Matlab^TM^2019 [23]. Each model produced a new array of values representing the PSG parameter. The correlation coefficient using Spearman [24] was evaluated between the predicted (model) and actual values (extracted from the PSG report).

(1) $$Predicted\;PSG\;parameter=a_{1}XY+a_{2}XZ+a_{3}YZ+a_{4}X+a_{5}Y+a_{6}Z+a_{7}$$

The number of models was then reduced based on their correlation with the actual PSG parameter and the percentage of overlap between different PSG parameter severity groups; the first 20 models with the highest correlation and the lowest overlap percentage were selected for the classification process; see [25] for more details on this step.

### 2.5. Classification and Prediction of PSG Parameters

Breathing sound signals are stochastic signals in nature, and the OSA disorder has many confounding factors that affect breathing sounds, which increases the heterogeneity and complexity of data. Therefore, it is required to have a classification process with multiple thresholds for each feature to overcome the complexity and heterogeneity; such a classification approach is possible in the random-forest (RF) algorithm [26]. In addition, RF does not require a normality assumption. Therefore, a classification process was performed using a two-class RF classifier with 1200 iterations using the selected threshold and the selected models. For each iteration, the classifier used 65% of the training/validation data for training and the other 35% for validation. Then, the validated classifier was used to classify the blind testing dataset. The input for the classifier was the predicted PSG parameter using the evaluated bilinear model; thus, it was a one-feature RF classifier. The models (three-, four-, five-feature combinations) that provided the validation and testing accuracies ≥70% and validation and testing sensitivities and specificities ≥50% were selected as the best models. Then, we combined the classifier outcomes of three models to find the best combinations covering most of the dataset while providing the highest classification performance. The final classification was based on the majority voting of each individual classifier; see [25] for more details on this section. The classification process was separately applied on the five data configurations, and then their results were averaged for each PSG parameter separately.

## 3. Results

The selected anthropometric (F1–F6) and sound features (F7–F79) for the analyses are listed in Table 1. The anthropometric characteristics of the study sample are presented in Table 2.

**Figure 4:**
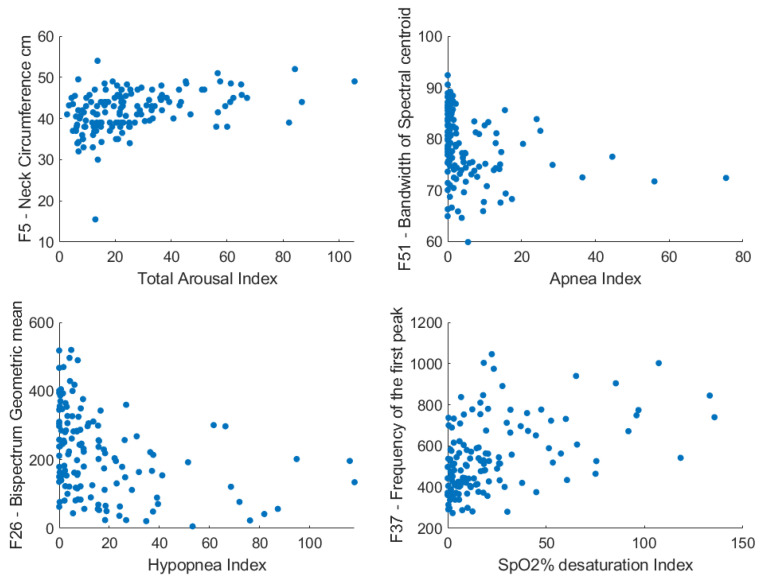
The scatter plots between four PSG parameters and the anthropometric and sound features with the highest correlation coefficients with them. F: feature label.

Table 3 shows the most significant and useful correlation coefficients (a maximum of 0.56) between the PSG parameters and anthropometric/sound features. Only those PSG parameters that resulted in high testing results are presented. Males had a higher number of apneic events than females. The increase in neck circumference and/or BMI was associated with an increase in the number of obstructive events and arousals. A high arousal index and apnea/hypopnea index were associated with an increase in the power of the high-frequency components of tracheal breathing sounds. Furthermore, a high oxygen desaturation index was associated with a high first resonance frequency in breathing sounds. Figure 4 shows the scatter plots of four PSG parameters with their correlated features.

**Figure 5:**
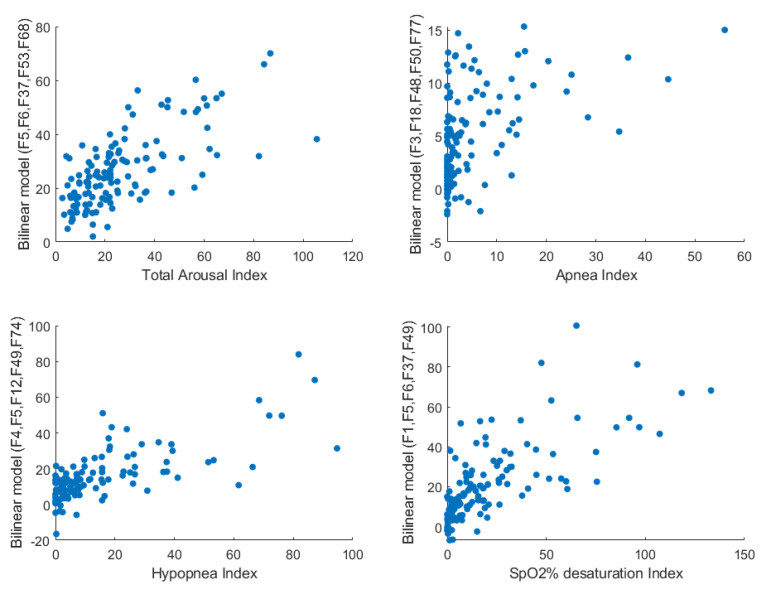
The scatter plots between four PSG parameters and the outcomes of the bilinear model. F: feature label.

Table 4 shows the bilinear model feature combinations with the highest overall correlation coefficients with the PSG parameters. Only the highest two model coefficients are represented in parentheses right to the relevant feature, and the bolded feature is the one with the highest contribution to the constructed model. Furthermore, F1 scores and testing classification accuracies of the blind testing dataset using the corresponding models to predict the PSG parameters are presented. The constructed models had correlation coefficients >0.8 with nine OSA severity parameters. The supine arousal index had a correlation coefficient of 0.8 with the F5-F6-F37-F46-F74 bilinear model, and the average power of high frequencies for the nose inspiratory signal was the main parameter in the bilinear model. The hypopnea index had a correlation coefficient of 0.82 with the F4-F5-F12-F26-F38 bilinear model, and neck circumference was the main parameter in the bilinear model. The testing classification accuracy and F1 score were 92% and 95%, respectively, for predicting AHI measured in the supine position with a threshold of 15. The scatter plots of the four previously mentioned PSG parameters with their highly correlated bilinear equation models are shown in Figure 5. These plots show the enhancement in correlation coefficients and linear relationships compared to those shown in Figure 4.

**Figure 6:**
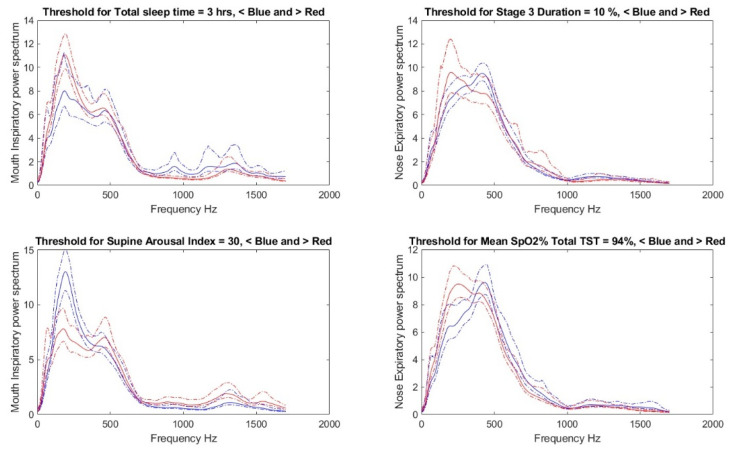
The average power spectrum of the signal recorded from mouth inspiration. Dotted lines represent the 95% confidence interval. Red and blue color curves represent the groups with more and less than the threshold, respectively.

Figure 6 shows the average inspiratory/expiratory power spectra during mouth/nose breathing of four PSG parameter groups. The corresponding sound spectra to each PSG parameter are represented using two curves (i.e., blue and red) with their 95% confidence intervals (dotted lines). The blue curve represents the average curves of individuals with the PSG parameters less than the selected threshold, while the red curve represents the average curves of individuals with the PSG parameters more than the selected threshold. There is a big gap in the range of 100–300 Hz between the mouth inspiratory power spectra of patients with a supine arousal index of more than 30 and less than 30.

On average, a healthy adults’ sleep duration is 5% in stage 1, 50% in stage 2, 20% in stage 3, and 20–25% in REM [18]. We used thresholds of 10% for stage 3 sleep as an indicator of abnormal sleep duration. The total arousal index threshold depends on age; it is 10 for young, 15–20 for middle-aged, and 25 for elders [19]. We used 25 as the threshold for predicting the total arousal index, as it had more separability in the power spectra of the training dataset (80% of the data). Furthermore, it has been mentioned that a normal mean SpO2% is between 93% and98% [20]; thus, we used 93–94% as a threshold of the beginning of an abnormal percentage. Moreover, de-SPO2 index is a value usually between 0 to 135, and it is highly correlated with AHI [21]; thus, we used 15 as the threshold for de-SPO2 index similar to an AHI of 15.

Table 5 shows the selected thresholds for the PSG parameters. The presented PSG parameters are the ones with average classification accuracies of more than 70% and average classification sensitivities and specificities of more than 60%. Table 5 also shows the average validation and blind testing classification accuracies, sensitivities, specificities, and F1 scores using the outcomes of the bilinear equations for the five dataset configurations. The proposed method resulted in a blind testing classification accuracy of up to 88.8%. Predicting the obstructive hypopnea index measured on the supine position with a threshold of 15 resulted in 81.6% blind testing accuracy with 86.1% validation accuracy and comparable and high F1 scores for both the validation and blind testing processes. In addition, many PSG parameters were predicted with reasonable and comparable accuracies, sensitivities, and specificities.

## 4. Discussion

The results of this study show the possibility to predict AHI and other OSA severity parameters with high precision during wakefulness by using only anthropometric information and five cycles of breathing sounds. Thus, the proposed methodology shows great potentials for a quick, informative, and highly reliable OSA screening tool.

Tracheal breathing sounds’ features showed significant correlations with some of the PSG parameters (Table 3). Feature 37 showed a high positive correlation with the total arousal index. This suggests that individuals with high arousal index have a high first frequency peak. The peaks at higher frequency represent stiffness, and that implies high UA dilator muscle activities [15]. Feature 47 was found to be high in individuals with a low average SpO2% during sleep. This indicates high power at low frequencies among those with high SpO2%; this is also congruent with our previous findings [15] as individuals with AHI < 15 have high power in the low-frequency components. Furthermore, this high power indicates a wide UA and, thus, low UA resistance.

As shown in Table 1, most of the selected sound features were extracted from inspiratory breathing; inspiration is an active process, hence, the UA muscles are active. This finding was expected because the UA muscle atrophy is one of the causes of the UA collapse leading to OSA. Thus, we expected the characteristic features of the breathing sounds in relation to OSA to be mostly selected during the inspiratory phase. This is also congruent with our previous studies [15,27], in which the characteristic breathing sounds in correlation with AHI were mostly selected from an inspiratory phase. Furthermore, PSG parameters were correlated with various sound features, which were extracted from linear and non-linear algorithms and from time and frequency domains. This indicates the complexity of the UA structure and the ability of breathing sound features to identify different UA characteristics.

Using models of bilinear equations resulted in better correlations with the PSG parameters than using only one-feature combinations (see Figure 5 and Table 4). Based on the selected thresholds and using the selected model combinations, we were able to reach up to 86.1% and 88.8% average validation and blind testing classification accuracy (VCA and TCA) with comparable sensitivities and specificities. Using five different data configurations and executing the entire process for each configuration ensured the unbiased reliability of the extracted models. The F1 scores, sensitivities, and specificities show the ability of the classifiers to have unbiased decisions. Therefore, the results demonstrate the ability to predict the PSG parameters with high confidence using combinations of anthropometric and breathing sounds features. The proposed method could be used as a fast-diagnosing tool that predicts several OSA severity parameters, and could benefit sleep physicians, anesthesiologists, and dentists.

Anthropometric parameters such as BMI, NC, age, etc. have shown their ability to screen for OSA; however, they provide low screening specificity. Nevertheless, as shown in this study, they had significant correlations with the attributes describing the OSA disorder evaluated by a PSG assessment. Sleep efficiency (TST/total time on bed) and stage 3 duration (representing deep sleeping) have been shown to decrease with age [18]. Additionally, average SpO2% and REM sleep duration were shown to decrease with obesity (increasing BMI) [28]. Our data (Table 3) are congruent with the above observations. In addition, our results showed that many event indices per hour during sleep, such as total arousal index, hypopnea index, total de-SaO2 index, etc., are positively correlated with NC. Furthermore, NC was selected as a significant feature in many models predicting several PSG parameters, which indicates its importance on the OSA screening process. Therefore, the outcomes confirm the relationships between OSA risk factors and the pathophysiology of the disorder.

In our previous works, as well as the work of other researchers [8,15,29,30,31], tracheal breathing sound features showed a high correlation with OSA severity in terms of AHI values. Investigating the power spectra for PSG threshold determination revealed some interesting observations. For example, the selected threshold for the apnea index in this study was 1, while it was 10 for the hypopnea index. This implies that an apneic event affects the UA much more significantly than a hypopnic event. Additionally, the selected supine arousal index threshold was 25, while it was 30 for the non-supine arousal index. The thresholds for hypopnea index and de-SPO2 index during non-REM sleep were lower than the threshold selected during REM sleep. Furthermore, the thresholds for obstruction indexes, in general, were higher when the patients were sleeping on their left side than those recorded when they were on their right side. These observations show a significant change in the UA structure in supine or right-side body positions and/or during non-REM sleep, and imply that the upper airway is at a higher risk if an obstruction event happens.

### Limitations and Future Work

The main limitation of this study was its sample size (*n* = 145), which, given the heterogeneity of the OSA population, is not enough to represent the whole OSA population. Future studies with a larger sample size are needed to replicate and validate the claims of this paper. The thresholds were mainly selected based on the separability between the two PSG parameter groups, while having a reasonable number of individuals in each group. Therefore, it is recommended to replace some of the selected thresholds with the ones that imply high health risks. The dataset could not be matched in terms of all of the mentioned anthropometric parameters due to the small sample size. Once we have a larger sample, we will investigate predicting each PSG parameter in different anthropometric groups, the same as the AWakeOSA analysis. Another limitation is that in this study, all the features were adopted from our previous study [8], and not extracted from visualizing the spectra of each PSG parameter. In future work, we will investigate the spectra for each PSG parameter separately to extract the most prominent features and assess whether those features would be much different from the current ones. Furthermore, the PSG reports did not have any information regarding average apnea or hypopnea events’ duration and average oxygen desaturation percentage duration during obstructive events. Once we resume our sleep studies in our sleep lab, we will consider recording them in our future studies, and investigate the possibility of their prediction.

## 5. Conclusions

Some PSG parameters, such as AHI, sleep duration, number of arousals, etc., are crucial to understand the pathophysiology of the OSA disorder and choose an appropriate treatment. Evaluating the parameters using simple means during wakefulness in a short time is an essential step towards an expeditious treatment; moreover, it will significantly help anesthesiologists who need to know the OSA status of their patients prior to surgery. This study is the first to investigate the prediction of the PSG parameters other than AHI during wakefulness using individuals’ anthropometric information and a few minutes of breathing sound recording. The promising results of this study for predicting the PSG parameters with meaningful features may pave the way for reducing the waiting lines and providing sleep clinics with a quick and reliable OSA screening tool.

## Figures and Tables

**Table 1:** Descriptions and details of the selected anthropometric and sound features.

FL	BM	Feature’s Definition
F1	-	Body mass index (BMI)
F2	-	Age
F3	-	Sex
F4	-	Weight
F5	-	Neck circumference (NC)
F6	-	Mallampati score (MpS)
F7	InsN	f1=350f2=525Mean of P(f)
F8	ExpM	f1=235f2=130Mean of P(f)− f1=1260f2=1410Mean of P(f)
F9	InsN	f1=275f2=115Mean of P(f)− f1=915f2=1240Mean of P(f)
F10	InsM	f1=75f2=1800 Frequency of the first peak of P(f)
F11	InsM	f1=75f2=1700Bandwidth of the spectral centroid of P(f)
F12	ExpM	f1=300f2=550Bandwidth of the spectral centroid of P(f)
F13	InsM	f1=0f2=600 Frequency of the first peak of P(f) using zero-crossing
F14	InsM	Total B(f,f) entropy
F15	InsM	f1=1200f2=1515First−order moment of the positive diagonal of B(f,f)
F16	InsM	f1=1200f2=1515First−order moment of the negative diagonal of B(f,f)
F17	ExpM	f1=130f2=235Second−order moment of the 0.5f−f line of B(f,f)
F18	InsN	Weight center of the positive diagonal of B(f,f)
F19	InsN	f1=250f2=355Mean of the positive diagonal of B(f,f)
F20	InsN	f1=250f2=355Weight center of B(f,f)
F21	InsN	f1=115f2=275First−order moment of the positive diagonal of B(f,f)
F22	InsN	f1=350f2=525Weight center of the negative diagonal of B(f,f)
F23	InsN	f1=245f2=350Mean of the slope of P(f)
F24	InsN	f1=100f2=350Bandwidth of the spectral centroid of P(f)
F25	InsM	Higuchi fractal dimension
F26	InsM	f1=140f2=270Geometric Mean of B(f,f)
F27	InsM	*Total* Weight center of B(f,f)
F28	InsM	f1=140f2=270First−order moment of the 2f−f line of B(f,f)
F29	ExpM	*Total* Weight center of B(f,f)
F30	InsN	f1=130f2=275Mean of B(f,f)
F31	InsN	f1=130f2=275Second−order moment of the positive diagonal of B(f,f)
F32	InsM	f1=130f2=230Mean of P(f)
F33	InsN	f1=130f2=280Mean of P(f)
F34	InsN	f1=240f2=340Mean of the slope of P(f)
F35	InsN	f1=80f2=350Bandwidth of the spectral centroid of P(f)
F36	InsN	f1=80f2=560Spectral centroid of P(f)
F37	InsM	f1=0f2=1800 Frequency of the first peak of P(f) using zero-crossing
F38	InsM	*Total* Weight center of the positive of diagonal B(f,f)
F39	InsM	f1=130f2=230First−order moment of the 2f−f line of B(f,f)
F40	InsM	f1=130f2=230Second−order moment of the 2f−f line of B(f,f)
F41	InsN	f1=130f2=280Mean of B(f,f)
F42	InsN	f1=130f2=280Mean of the negative diagonal of B(f,f)
F43	InsN	f1=130f2=280Second−order moment of the negative diagonal of B(f,f)
F44	ExpM	f1=375f2=485Mean of P(f)
F45	InsN	f1=360f2=500Mean of P(f)
F46	InsN	f1=1010f2=1250Mean of P(f)
F47	InsN	f1=270f2=370Mean of the slope of P(f)
F48	ExpN	f1=450f2=550Mean of the slope of P(f)
F49	ExpM	f1=200f2=600Bandwidth of the spectral centroid of P(f)
F50	InsN	f1=440f2=620Spectral centroid of P(f)
F51	ExpN	f1=300f2=600Bandwidth of the spectral centroid of P(f)
F52	InsM	f1=390f2=510Weight center of the 2f−f line of B(f,f)
F53	ExpM	f1=270f2=390Weight center of the negative diagonal of B(f,f)
F54	ExpM	f1=270f2=390Weight center of the positive diagonal of B(f,f)
F55	ExpN	f1=450f2=550Mean of the negative diagonal of B(f,f)
F56	InsN	f1=355f2=510Mean of P(f)/ f1=820f2=1070Mean of P(f)
F57	InsN	f1=250f2=350Mean of the slope of P(f)
F58	InsM	f1=90f2=550Spectral skewness of P(f)
F59	InsN	f1=0f2=550 Frequency of the first peak of P(f) using zero-crossing
F60	InsM	f1=120f2=250Mean of B(f,f)
F61	InsM	f1=120f2=250First−order moment of the positive diagonal of B(f,f)
F62	InsN	f1=355f2=510Weight center of the negative diagonal of B(f,f)
F63	InsN	f1=820f2=1070Second−order moment of the 2f−f line of B(f,f)
F64	ExpN	f1=100f2=600Weight center of the 0.5f−f line of B(f,f)
F65	InsN	f1=395f2=520Mean of P(f)
F66	InsM	f1=60f2=600 Frequency of the peak of P(f)
F67	InsN	f1=150f2=550Spectral centroid of P(f)
F68	InsM	f1=60f2=600Weight center of the 0.5f−f line of B(f,f)
F69	InsM	f1=60f2=600Second−order moment of the 2f−f line of B(f,f)
F70	InsN	f1=395f2=520Weight center of the positive diagonal of B(f,f)
F71	InsN	f1=395f2=520Mean of the negative diagonal of B(f,f)
F72	ExpN	f1=100f2=600Weight center of the negative diagonal of B(f,f)
F73	InsM	f1=130f2=230Mean of P(f)/ f1=1260f2=1460Mean of P(f)
F74	InsM	f1=1090f2=1460First−order moment of the positive diagonal of B(f,f)
F75	InsM	f1=1260f2=1460First−order moment of the positive diagonal of B(f,f)
F76	InsM	f1=1090f2=1460Weight center of the 0.5f−f line of B(f,f)
F77	InsM	f1=1260f2=1460First−order moment of the 0.5f−f line of B(f,f)
F78	ExpM	f1=1260f2=1410First−order moment of the negative diagonal of B(f,f)
F79	InsN	f1=300f2=1600First−order moment of the 0.5f−f line of B(f,f)

Ins/Exp: inspiration/expiration, M/N: mouth/nose, mean: arithmetic mean, *P(f)*: the power spectrum, *B(f, f)*: the bispectrum, *f*: frequency, FL: feature label, and BM: breathing maneuver.

**Table 2:** Anthropometric information characteristics of the study.

	Non-OSA (AHI < 15) *n* = 80	OSA (AHI ≥ 15) *n* = 65
AHI, events/h, median (IQR)	2.9 (0.33–6.68)	29.4 (20.1–57.3)
Age, years, median (IQR)	50 (41–57)	51 (43–59)
Sex, n (%)		
Female	43 (53.8)	17 (26.2)
Male	37 (46.3)	48 (73.8)
BMI, kg/m^2^, median (IQR)	31.2 (26.9–35.7)	34.8 (30.2–39.8)
NC, cm, mean (SD)	39.87 (4.98)	44.37 (3.74)
MpS, n (%)		
I	45 (56.3)	15 (23.1)
II	21 (26.3)	23 (35.4)
III	11 (13.8)	19 (29.2)
IV	3 (3.8)	8 (12.3)

OSA is obstructive sleep apnea, NC is neck circumference, MpS is Mallampati score, IQR is interquartile range, and SD is standard deviation. AHI is Apnea-hypopnea index; BMI is body mass index.

**Table 3:** The spearman correlation coefficients between the anthropometric/sound feature and the PSG parameters.

PSG#	PSG Parameter	Anthropometric and Sound Feature	Correlation Coefficient
PSG1	Stage 3 Duration %	F2	−0.35
PSG2	EEG Total Arousal Index	F11/F37/F8	0.35/0.33/−0.31
PSG3	Total Arousal Index	F5/F11/F37	0.41/0.35/0.33
PSG4	Supine Arousal Index	F5/F37/F46/F53	0.46/0.36/0.31/0.31
PSG5	Non-Supine Arousal Index	F1/F4/F10/F69	0.37/0.36/0.36/0.36
PSG6	Supine Sleep %	F1/F17	−0.29/0.29
PSG7	Apnea Total Index	F3/F5/F51	−0.5/0.41/−0.38
PSG8	Apnea Non-REM Index	F3/F5/F49	−0.51/0.46/−0.39
PSG9	Apnea Total Index (No Central)	F3/F5/F51	−0.44/0.38/−0.33
PSG10	Apnea Non-REM Index (No Central)	F3/F5/F49/F37	−0.45/0.43/−0.37/0.36
PSG11	Hypopnea Total Index	F5/F26/F4/F37/F49	0.54/−0.45/0.41/0.4/−0.4
PSG12	Hypopnea REM Index	F31	−0.34
PSG13	Hypopnea Non-REM Index	F5/F4/F37/F57	0.53/0.41/0.41/0.4
PSG14	Hypopnea Total Index (No Central)	F5/F32/F4/F49	0.54/−0.43/0.41/−0.4
PSG15	Hypopnea REM Index (No Central)	F1/F20/F4/F6	0.3/0.28/0.27/0.26
PSG16	Hypopnea Non-REM Index (No Central)	F5/F4/F37/F49	0.53/0.41/0.41/−0.4
PSG17	Supine Sleep Obs. Apnea	F5/F3/49	0.43/−0.43/−0.36
PSG18	Supine Sleep Obs. Hypopnea	F5/F26/F49	0.51/−0.38/−0.37
PSG19	Supine Sleep A + H (with central)	F5/F37/F57/F10	0.6/0.41/0.38/0.36
PSG20	Supine Sleep A + H (without central)	F5/F57/F26/F49	0.55/0.42/−0.4/−0.41
PSG21	Left-side Sleep Obs. Hypopnea	F5/F1/61/F32	0.45/0.42/−0.38/−0.38
PSG22	Left-side Sleep A + H (with central)	F5/F1/F32/F4	0.45/0.4/−0.39/0.36
PSG23	Left-side Sleep A + H (without central)	F1/F32/F4	0.4/−0.4/0.37
PSG24	Right-side Sleep Obs. Hypopnea	F26/F69/F1/F38	−0.44/−0.43/0.41/0.41
PSG25	Right-side Sleep A + H (with central)	F8/F5/F67/F27	−0.43/0.43/0.41/0.4
PSG26	Right-side Sleep A + H (without central)	F21/F26/F5/F8/F9	−0.43/−0.44/0.42/−0.43/−0.42
PSG27	Total AHI (With Central)	F5/F21/F25/F54	0.56/−0.41/0.4/0.4
PSG28	Total AHI (Without Central)	F5/F37/F57/F49	0.54/0.43/0.43/−0.42
PSG29	De-SpO2 Index Total	F5/F60/F37/F1	0.56/−0.44/0.42/0.4
PSG30	De-SpO2 Index Non-REM	F5/F4/F21/F37	0.55/0.43/−0.41/0.42
PSG31	De-SpO2 Index REM	F1/F5/F8/F23	0.41/0.39/−0.38/0.38
PSG32	Mean SpO2% Total TST	F1/F47/F4	−0.4/−0.39/−0.36
PSG33	Mean SpO2% Supine	F47	−0.32
PSG34	Mean SpO2% Non-Supine	F1/F4/F47/F37	−0.47/−0.43/−0.4/−0.39
PSG35	REM Latency/TST	F28/F29	−0.29/0.25
PSG36	Wake after sleep Onset/TST	F2	0.39

F: feature label, BMI: body mass index, NC: neck circumference, A+H: apnea and hypopnea, and REM: rapid eye movement. PSG#: PSG parameter number. SpO2: Oxygen saturation. De-SpO2: Oxygen desaturation. EEG: Electroencephalogram. TST: total time of sleep.

**Table 4:** The correlation coefficients between the PSG parameters and the outcomes of the bilinear model for the entire dataset, as well as their blind testing F1 scores and classification accuracies.

PSG#	Feature Combination	CC	F1-Score	ACC%
PSG1	F2 **F3**(0.31) F46(−0.14) F74 F77	0.63	0.63	63
PSG2	F8(7.3) F12 F46(110) F49 F56	0.7	0.82	80
PSG3	F5(−31) **F6**(246) F37 F53 F68	0.74	0.76	81
PSG4	F5 F6(−21) F37 **F46**(86) F74	0.8	0.62	64
PSG5	F1 F10 F49(0.73) **F56**(−3.2) F72	0.71	0.62	74
PSG6	F2 F4 F33(0.07) F35 **F46**(2.3)	0.64	0.64	64
PSG7	F3(106) F18 **F48**(−1367) F50 F77	0.75	0.74	71
PSG8	F3(8.4) F37 **F48**(−562) F49 F51	0.72	0.67	71
PSG9	F3 F19 F46(30.7) **F48**(−342) F77	0.69	0.6	67
PSG10	F3(30.7) **F6**(64.4) F19 F53 F78	0.72	0.63	68
PSG11	F4(2.6) **F5**(13.4) F12 F26 F38	0.82	0.83	82
PSG12	F4(−5.5) F5 **F12**(−14) F49 F74	0.72	0.67	79
PSG13	F4 **F5**(17.1) F11 F49(−5.5) F52	0.81	0.6	62
PSG14	**F1**(−7.2) F4(5.5) F5 F12 F15	0.76	0.78	78
PSG15	F1(−0.96) F29 F30 **F49**(−1.3)	0.59	0.6	78
PSG16	F5 F6(36.3) F10 **F46**(288) F53	0.78	0.8	79
PSG17	F3 F46(11.1) **F57**(79.1) F66 F77	0.75	0.73	77
PSG18	F4 **F5**(9.9) F10 F17 F49(2.1)	0.79	0.62	71
PSG19	F5(3.1) F38 **F57**(−730) F68 F71	0.79	0.95	92
PSG20	F5(32.3) **F6**(−99) F11 F50 F74	0.83	0.86	85
PSG21	F4(3.9) **F5**(−10.8) F24 F42	0.64	0.78	74
PSG22	F4 F5(−3.3) **F6**(33.48) F32 F66	0.73	0.67	69
PSG23	F1 **F6**(473) F32(−80.5) F49 F53	0.73	0.63	67
PSG24	**F6**(−15.3) F8(3.9) F10 F38 F41	0.81	0.77	73
PSG25	F5(−76) **F6**(113) F19 F54 F77	0.79	0.67	60
PSG26	F6 F9(−18) F26 F38 **F46**(−23.5)	0.84	0.73	72
PSG27	F5(−161) 21 **F25**(−2550) F38 F51	0.81	0.67	75
PSG28	F5 F11 F25(−1889) **F57**(−4046) F71	0.8	0.63	67
PSG29	F1 F5(4.4) **F6**(−8.4) F37 F49	0.77	0.63	73
PSG30	F5(12.73) F21 F53 **F65**(−89.5) F77	0.78	0.84	80
PSG31	**F6**(44) F16 F29 F30 F65(24.7)	0.8	0.89	92
PSG32	**F1**(−0.005) F24(0.0009) F29 F51	0.65	0.62	58
PSG33	F18 F28 F29 **F47**(0.15) F68(−0.0004)	0.63	0.72	68
PSG34	**F1**(0.0157) F2(−0.006) F31 F37 F53	0.71	0.8	72
PSG35	**F1**(0.22) F2 F28 F32(−0.13) F36	0.63	0.74	74
PSG36	F2(−0.75) F22 F39 **F46**(1.78) F76	0.55	0.7	65

F: feature label, PSG#: PSG parameter number, CC: correlation coefficient with the outcomes of the bilinear model, and ACC: testing classification accuracy. Feature coefficient in the model is represented in the parentheses, and bolded features are the ones with the highest contribution to the constructed model.

**Table 5:** The average validation and testing accuracies for classifying data around the selected threshold using the outcomes of the bilinear models.

PSG#	Threshold	VF1	VCA%	VSpec%	VSens%	TF1	TCA%	TSpec%	TSens%
PSG1	10%	0.66	80.7	88.9	61.9	0.73	78.2	80.1	73.8
PSG2	15	0.80	75.3	69.1	79.2	0.83	81.6	78.0	83.8
PSG3	25	0.65	74.6	83.9	60.6	0.66	73.6	81.6	61.7
PSG4	30	0.75	76.8	78.7	74.5	0.72	73.4	73.7	73.7
PSG5	20	0.70	76.2	81.8	67.9	0.76	84.7	97.8	63.3
PSG6	30%	0.79	74.9	63.1	83.7	0.75	72.4	76.4	70.5
PSG7	1	0.81	80.8	80.6	81.1	0.83	82.9	82.1	84.0
PSG8	1	0.79	82.3	88.7	74.1	0.73	80.7	85.4	70.1
PSG9	3.5	0.70	83.4	90.4	66.6	0.76	85.6	89.7	78.1
PSG10	0.5	0.76	77.9	80.7	74.8	0.79	80.6	79.6	82.6
PSG11	10	0.78	83.0	88.5	75.0	0.74	80.4	82.2	76.4
PSG12	20	0.70	77.8	84.6	67.0	0.68	79.5	91.3	60.6
PSG13	7.5	0.83	84.5	86.7	81.9	0.78	80.4	80.1	80.2
PSG14	5	0.87	82.8	66.9	91.6	0.90	88.8	82.5	94.7
PSG15	20	0.67	76.2	82.0	66.3	0.68	80.6	89.6	62.5
PSG16	5	0.83	80.7	77.2	83.5	0.83	82.6	77.7	90.3
PSG17	1	0.77	77.1	80.1	74.1	0.77	79.9	85.6	74.5
PSG18	15	0.86	86.1	85.3	86.9	0.78	81.6	82.1	79.8
PSG19	15	0.87	85.7	83.9	86.9	0.82	79.7	71.8	86.3
PSG20	20	0.84	83.4	80.6	85.8	0.81	81.4	79.4	83.4
PSG21	2.5	0.76	78.8	82.1	74.1	0.78	80.4	78.5	84.8
PSG22	7	0.76	84.4	92.1	70.3	0.71	80.2	85.9	69.8
PSG23	4	0.74	80.1	87.1	69.6	0.75	79.5	78.0	79.2
PSG24	1	0.84	81.6	80.3	82.6	0.82	80.4	77.5	82.5
PSG25	0.5	0.87	82.5	71.9	88.2	0.91	88.4	94.3	86.9
PSG26	0.5	0.86	82.2	75.6	86.1	0.87	83.7	85.0	84.2
PSG27	15	0.77	78.7	81.2	75.8	0.78	82.4	85.0	79.3
PSG28	15	0.76	80.5	87.1	71.7	0.75	80.5	83.9	74.5
PSG29	15	0.75	77.2	81.1	72.8	0.78	82.0	84.5	78.7
PSG30	5	0.87	83.0	70.7	89.5	0.89	86.5	77.9	91.6
PSG31	25	0.78	83.3	91.5	71.5	0.77	82.5	83.9	80.0
PSG32	94%	0.72	70.5	68.7	72.0	0.74	71.5	68.6	75.4
PSG33	94%	0.69	72.3	77.0	66.6	0.78	77.8	74.7	81.9
PSG34	93%	0.88	81.8	60.1	90.9	0.92	87.8	77.1	92.3
PSG35	0.45	0.78	72.4	64.3	77.2	0.88	86.5	100	80.6
PSG36	0.15	0.78	72.4	60.0	79.8	0.79	74.1	63.9	80.8

F: feature label, PSG#: PSG parameter number, V: validation, T: blind testing, CA: classification accuracy using the outcomes of the bilinear models, F1: F1 score, Spec: specificity, and Sens: sensitivity.

## Data Availability

Data are available for interested researchers from this page: https://home.cc.umanitoba.ca/~mousavi/ftp/Data/.

## Appendix A

**Table A1:** Shows the extracted PSG parameters from the PSG report.

PSG Parameter
Total sleep time TST
Sleep Efficiency
Total Awakenings
Total Awakenings Index
Stage 1 Duration %
Stage 2 Duration %
Stage 3 Duration %
Stage 4 Duration %
REM Duration %
Respiratory-Related Total Arousal Index
PLM-Related Total Arousal Index
EEG Total Arousal Index
Total Arousal Index
NREM Total Arousal Index
REM Arousal Index
Supine Arousal Index
Non-Supine Arousal Index
Total Arousal + Awakenings Index
Supine Sleep %
Prone Sleep %
Left-Side Sleep %
Right-Side Sleep
Apnea Total Index
Apnea REM Index
Apnea Non-REM Index
Apnea Total Index (No Central)
Apnea REM Index (No Central)
Apnea Non-REM Index (No Central)
Hypopnea Total Index
Hypopnea REM Index
Hypopnea Non-REM Index
Hypopnea Total Index (No Central)
Hypopnea REM Index (No Central)
Hypopnea Non-REM Index (No Central)
Supine Sleep Obs. Apnea
Supine Sleep Mix. Apnea
Supine Sleep Cen. Apnea
Supine Sleep Obs. Hypopnea
Supine Sleep A+H (with central)
Supine Sleep A+H (without central)
Prone Sleep Obs. Apnea
Prone Sleep Mix. Apnea
Prone Sleep Cen. Apnea
Prone Sleep Obs. Hypopnea
Prone Sleep A+H (with central)
Prone Sleep A+H (without central)
Left-side Sleep Obs. Apnea
Left-side Sleep Mix. Apnea
Left-side Sleep Cen. Apnea
Left-side Sleep Obs. Hypopnea
Left-side Sleep A+H (with central)
Left-side Sleep A+H (without central)
Right-side Sleep Obs. Apnea
Right-side Sleep Mix. Apnea
Right-side Sleep Cen. Apnea
Right-side Sleep Obs. Hypopnea
Right-side Sleep A+H (with central)
Right-side Sleep A+H (without central)
Total AHI (With Central)
Total AHI (Without Central)
De-SpO2 Index Total
De-SpO2 Index Non-REM
De-SpO2 Index REM
Mean SpO2% Wake
Mean SpO2% NREM
Mean SpO2% REM
Mean SpO2% Total TST
Mean SpO2% Supine
Mean SpO2% Non-Supine
SpO2% ≤ 85% percentage of TST
Sleep Onset
REM Latency
REM Latency Less wake
Wake after sleep Onset
Sleep Onset/TST
REM Latency/TST
REM Latency Less wake/TST
Wake after sleep Onset/TST

Table A2 shows the best classification results for each PSG parameter. Furthermore, it shows the validation and blind testing classification accuracies for one of the data configurations.

**Table A2:** The validation accuracy for classifying data around the selected threshold using the outcomes of the bilinear models for each of the three severity groups separately.

PSG Parameters	Threshold	VCA_2_Total_	VCA_2_<15_	VCA_2_>15_	TCA_2_Total_	TCA_2_<15_	TCA_2_>15_
Stage 3 Duration %	10%	85.71%	85.00%	84.44%	75.00%	83.33%	25.00%
Total Arousal Index	25	70.87%	72.88%	63.64%	80.95%	85.00%	0.00%
Apnea Total Index	1	79.46%	75.00%	84.09%	82.61%	81.82%	100.00%
Apnea Non-REM Index	1	83.33%	86.57%	78.72%	90.00%	92.59%	66.67%
Hypopnea Total Index	10	90.09%	96.83%	81.25%	85.71%	82.61%	100.00%
Hypopnea Non-REM Index	7.5	81.74%	81.82%	75.51%	85.71%	80.00%	100.00%
Hypopnea Total Index (No Central)	5	83.04%	79.03%	86.00%	93.33%	91.67%	100.00%
Supine Sleep Obs. Apnea	1	75.24%	70.97%	79.07%	86.96%	86.36%	100.00%
Supine Sleep Obs. Hypopnea	15	81.31%	80.65%	82.22%	77.78%	76.19%	83.33%
Supine Sleep A+H (with central)	15	86.67%	86.89%	86.36%	88.89%	86.96%	100.00%
Total AHI (With Central)	15	83.48%	89.23%	76.00%	86.67%	87.50%	83.33%
Total AHI (Without Central)	15	86.84%	93.75%	76.00%	85.71%	85.71%	85.71%
De-SpO2 Index Total	15	81.42%	84.13%	74.00%	92.86%	100.00%	75.00%
Mean SpO2% Total TST	94%	69.09%	63.93%	75.51%	72.00%	68.42%	83.33%
Mean SpO2% Supine	94%	81.40%	78.00%	80.56%	81.25%	91.67%	50.00%
Mean SpO2% Non-Supine	93%	86.09%	87.10%	84.91%	76.19%	72.22%	100.00%
Wake after sleep Onset/TST	0.15	73.53%	67.80%	76.74%	69.23%	66.67%	50.00%

V: validation, T: blind testing, CA_2_: classification accuracy using the outcomes of the second-order models, Total: all data, < 15: group of AHI < 15, and > 15: group of AHI > 15.
